# Book Notices

**Published:** 1881-01-20

**Authors:** 


### *BOOK NOTICES.*

A COMPENDIUM OF MODERN PHARMACY AND DRUGGIST'S FORMULARY: Containing the recent methods of manufacturing and preparing Tinctures, Fluid Extracts, Perfumery, Extracts, Emulsions, Toilet Articles, Wines and Liquors; also, Physician's Prescriptions, liniments, pills, powders, ointments, syrups, antidotes to poison, weights and measures, and miscellaneous information indispensible to the Pharmacist, by Walter B. Kilnner, Pharmacist, Springfield, Ill., 1880.

This is a work of 478 octavo pages, neatly and elegantly gotten up. It is certainly a very valuable collection of formular and pharmaceutical preparations, especially suited to the prescriptionists and pharmacists. We are much pleased with it. See advertisement.

COMPENDIUM OF MICROSCOPICAL TECHNOLOGY: A guide to Physicians and Students in the use of the microscope, and in the preparation of Histological and Pathological specimens, by Carl Seiler, M. D., late director of the Microscopical and Biological section of the Academy of Natural Sciences of Philadelphia; Curator of the Pathological Society; Pathologist and Microscopist to the Presbyterian Hospital, etc., etc.

This work is especially adapted to a beginner in microscopic study, and is plain, practical and admirably gotten up for the purposes in view.

MEDICAL HERESIES, HISTORICALLY CONSIDERED: A series of critical essays on the Origin and Evolution of Sectarian Medicine, embracing a Special Sketch and review of Homœopathy, past and present, by Gonzales C. Smythe, A. M., M. D., Professor of the Practice of Medicine, Central College of Physicians and Surgeons, Indianapolis, Member of American Medical Association, etc., Philadelphia. Pressley Blakiston, 1012 Walnut street, 1880.

A very interesting work of 228 pages, giving a condensed history of the evolution of medicine.

MINOR SURGICAL GYNECOLOGY: A Manual of Uterine diagnosis and the lower technicalities of gynecological practice, for the use of the advanced student and general practitioner, by Paul F. Munde, M. D., Prof. Gynecology in Dartmouth Medical College, Obstetric Surgeon to the Maternity Hospital, New York; Physician for Diseases of Women to the out-door department of Mt. Sinai Hospital, and late Assistant Surgeon to the New York Woman's Hospital, etc., etc., with 300 illustrations. New York, William Wood & Co., 27 Great Jones St, 1880. W. B. Dalston, Agt., Atlanta, Ga.

This is a work of 380 octavo pages, well gotten up, timely in its objects, and eminently practical. The author remarks truly, "That many an error may be avoided. and many a manipulation rendered easy for physician and patient, if the sources of possible error and the details of the manipulation be clearly laid down before the operator. With this object, the book has been prepared. The author has, perhaps, as nearly accomplished his object as is preaticable for it to be done, and the work before us, we opine, will be found more satisfactory in the simplicity of its descriptions and the clearness of detail, than any work yet issued in this department.

YELLOW FEVER, ITS SHIP ORIGIN AND PREVENTION:—By Robert B S. Hargis, M. D., Pensacola, Fla. D. G. Brinton, M. D., publisher, 115 South Seventh street, Philadelphia.

A work of 76 octavo pages. The views of the writer are forcibly expressed, and are well worthy of perusal by every one who is interested in the important subject of which he treats.

